# Scientific Misunderstanding: Notes for the Bedeviled

**DOI:** 10.1007/s42113-026-00337-0

**Published:** 2026-09-15

**Authors:** David Kellen, Mikhail S. Spektor

**Affiliations:** 1 Department of Psychology, Syracuse University, Syracuse, NY USA; 2 College of Arts and Sciences, VinUniversity, Hanoi, Vietnam; 3 Department of Psychology, University of Warwick, Coventry, UK

**Keywords:** Philosophy of science, Inference, Explanation, Causality, Statistical paradoxes, Regression to the mean

## Abstract

In this paper, we critically examine the essay by Shiffrin, Stigler, and Keil (2026) on scientific understanding. We argue that, despite its uncontroversial moral message, the paper largely fails to articulate a compelling thesis. The central notion of an “illusion of understanding” is left insufficiently specified and supported by ill-suited examples. The analysis further rests on a narrow conception of explanation that largely equates understanding with causal claims. When discussing linear regression and related examples (e.g., Simpson’s Paradox), the essay also fails to clarify what is actually at stake. Its treatment of regression to the mean, in particular, is misconceived. We conclude by suggesting that discussions of scientific understanding would benefit from a model-hierarchical perspective.

**Supplementary Information:**

The online version contains supplementary material available at https://doi.org/10.1007/s42113-026-00337-0.

In reading the essay by Shiffrin et al. (2026), we took the general message to be that scientific investigations, like any other human endeavor, are fraught with risk, limits, error, and uncertainty. While unobjectionable and worthy of regular reminding, this observation is too general to be of particular use. The essay’s ultimate contribution must therefore lie not in its general moral message but in the specific analyses and examples marshaled in its support. It is here, however, that the essay proves uneven. This abridged critique^1^ is an attempt to shed light on some of the points at the heart of the essay.

## Conceptual Ambiguities and their Concrete Equivocations

From the outset, the essay deliberately leaves the meanings of key terms such as ‘understanding,’ ‘explanation,’ and ‘causality’ ambiguous enough to accommodate common parlance. Unfortunately, this attempt at ecumenism leaves unclear what qualifies as an “illusion of understanding,” how it differs from legitimate knowledge, and how it departs from the familiar notion of misunderstanding. Put plainly, there is no discernible distinction between harboring an illusion and simply being wrong.

The consequences of this conceptual ambiguity appear in the examples offered to illustrate how a formal model may generate accurate predictions while remaining poorly understood or even misleading. We are given Newton’s incorrect explanation of a dice puzzle (see Stigler, 2006), then Quantum Field Theory, and finally systems such as ChatGPT. Individually the examples strike us as inadequate; together, we fail to see how they form a coherent picture.

The Newton episode concerns a one-off problem and shows only that a correct calculation can accompany a defective verbal gloss. Quantum Field Theory, though mathematically demanding, bears no obvious relation to the clarity of its concepts. And while large language models such as ChatGPT exhibit striking emergent behavior, it remains unclear what they are theories or models *of*, even when granting the whole tools-to-theories arc (Gigerenzer, 1991). More troubling is the implication that Newton’s episode identifies a pathology of inquiry. Newton’s first attempt can be seen as the opening move in the kind of dialectical process described by Lakatos in *Proofs and Refutations* (1976), where naive conjectures are iteratively repaired or abandoned in response to assumptions and counterexamples. Taking the essay at face value, it is not clear how this process of inquiry is to be seen as anything more than a carousel of illusions.

## When Because has No Cause

Despite the advertised conceptual ecumenism, the treatment of explanation is strikingly orthodox, with the essay repeatedly invoking causality and at times treating ‘theory’ and ‘model’ as synonymous with a causal account^2^, making clear that causal claims sit high on the authors’ hierarchy of explanatory virtues. Granting that much thinking about scientific explanation takes place inside the Temple of Salmon (Salmon, 1984), this framing risks misunderstanding. For one, it downplays the contemporary standing of non-causal explanations such as renormalization-group arguments, which locate physical phenomena within constraints that render alternatives impossible or unstable (see Morrison, 2018; Lange, 2016; Reutlinger, 2014; Reutlinger & Saatsi, 2018).

Privileging causal accounts this strongly also risks overlooking cases in which they are ill-posed and conducive to misunderstanding. For instance, psychological research offers many cases in which (causal) explanations collapse into mere conditions of intelligibility when explanandum and explanans are not logically independent, thereby insulating the explanatory claim from empirical challenge (Greve, 2001; Wallach & Wallach, 1994, 1998).

A related confusion appears to be present in the essay’s discussion of explanatory depth, at times equated with the accumulation of “layers of mechanistic details.” In its discussion of monarch-butterfly migration, the essay juxtaposes accounts of the transmission of location information across generations with studies of the molecular basis of navigational signals and wonders which of these accounts is “deeper.” This framing suggests a difficulty in preserving the logic of part–whole relations (Bennett & Hacker, 2022). The butterfly’s migratory capacity is a property *of the organism*, not of its molecular components. Explaining the biological basis of that capacity is *not* the same as explaining the capacity itself or its exercise (Hacker, 2011, Chap. 4; Kenny, 1989, Chap. 5). Treating additional mechanistic detail as explanatory depth risks confusing causal enablement with constitutive explanation and mistaking a shift in explanatory level for a gain in understanding (Bennett & Hacker, 2022; Trigg & Kalish, 2011).

## Sleights of Our Own Hand

We now turn to the essay’s analysis of linear regression and related paradoxes. The essay notes that many investigations begin by examining relationships among observed variables, making linear regression a natural first step. It then warns that such models represent only one possible description of the data and that their output admits multiple interpretations (e.g., causal). These observations are taken to show that the linear regression model is more difficult to understand than it appears, a point illustrated through examples such as Simpson’s and Lord’s paradoxes. Simpson’s Paradox occurs when a pattern observed in aggregated data reverses once the data are stratified by a third variable (Simpson, 1951). Lord’s Paradox concerns situations in which conclusions about change over time depend on whether one statistically controls for baseline differences (Lord, 1967).

In our view, this line of argument misidentifies the source of the difficulty. The regression model is not particularly obscure. It simply describes how the expected value of one variable varies as a linear function of specified covariates. The real difficulty lies in researchers’ expectations about what the model should deliver.

One such expectation is that the model should adjudicate among competing interpretations of its results. It does not. Interpretations, causal or otherwise, depend on substantive considerations *external to the model* and are legitimate targets of scrutiny, debate, and disagreement—none of which warrant the conclusion that illusions of understanding are at work.

Another mistaken expectation is that the model reveals free-floating facts about the variables involved. In reality, every regression carries an implicit weighting scheme. In pooled analyses, observations are weighted by their frequency in the sample; once the data are stratified, the same model yields a collection of conditional relationships, each weighted by the frequencies within its respective stratum. These summaries answer different questions and coexist without friction. When the weights implicit in the pooled analysis differ from those assumed when interpreting the stratified results, reversals à la Simpson are to be expected (see Gong & Meng, 2021).

A similar misunderstanding appears in discussions of Lord’s Paradox. The essay rather amusedly endorses the “uncorrected” comparison favored by Statistician 1, pointing to regression to the mean as a decisive argument against what it regards as a nonsensical alternative. But the statisticians’ conclusions are not contradictory, and neither is intrinsically nonsensical. They are simply alternative summaries of the same joint distribution constructed under different aggregation rules. One preserves the observed composition of the groups; the other evaluates the groups under a common baseline distribution. As with Simpson’s Paradox, given that groups typically differ in their initial composition, there is little reason to expect the resulting summaries to coincide. The real misunderstanding lies in the assumption that they should. In modern causalspeak, one summary corresponds to a *total effect*, the other to a *direct effect* adjusting for the mediating role of baseline weight (for a detailed discussion, see Pearl, 2016). In short, each of these summaries or effects answers a different, precise question; *they are not rival answers to a single vague one*. Determining which summary or effect is appropriate depends on the pertinent question being asked and other subject-matter considerations about how the variables relate (e.g., Miller & Chapman, 2001), the prevalence of measurement error (e.g., Pedersen et al., 2025; Tennant et al., 2023), and the handling of control conditions, or their absence (see Holland & Rubin, 1983; Wainer, 1991). In the Supplemental Materials, we show how Lord’s Paradox, along with the variable-reversal effect emphasized in the essay, can emerge from a single, coherent process account. We also show that the regression-to-the-mean account proposed in the essay operates under stringent constraints that press it toward implausible claims about measurement reliability.

## The Distribution Did It, Your Honor

Beyond its role in the discussion of Lord’s words, the essay gives separate treatment to regression to the mean, describing it as a long misunderstood “statistical phenomenon that occurs universally when two paired measures are not perfectly correlated” and warning that scientists often produce “causal accounts for data patterns that are produced by regression to the mean rather than causal mechanisms.” While this diagnosis gestures toward a genuine risk, it perpetuates a misunderstanding of what regression to the mean involves. As Clarke et al. (1960) put it: “To say without further qualification that test scores have changed ‘because of regression’ gives no greater information than to state that they have changed because they have changed.”

The essay defines regression to the mean by way of a conditional pronouncement: “if a member of one pair of values is extreme (in either direction), its partner would be expected to be less extreme.” The difficulty with this formulation is that it attributes a directional change to an individual case on the basis of its relation to a group-level abstraction. The individual is thus said to “move” toward the mean not because anything has happened to them, but because of how they have been situated within a particular reference class. Returning to the Lord, the same male student is expected to gain or lose weight depending on whether the comparison uses a mixed-sex or male-only mean.

Regression to the mean is not a tendency of individuals but a property of certain bivariate distributions. Although it holds for familiar cases such as the Gaussian distribution, it is not universal. Some distributions exhibit the opposite pattern—regression *away* from the mean (Maraun et al., 2011; Schwarz & Reike, 2018). For instance, this property arises in random samples drawn from the family of Ex-Gaussian distribution, often used to model response times (see Schwarz & Reike, 2018). Whether regression toward or away from the mean appears in a given dataset is therefore an empirical matter. More importantly, such a statistical property cannot itself serve as an explanation. Explanations of individual-level events must be framed in terms of claims at that level, rather than by invoking relationships defined over populations (Lamiell, 1981, 2019).

## Concluding Thoughts

Because the essay’s moral message is so uncontroversial, readers may overlook substantive problems in the analysis. In attempting to map illusions of understanding in the sciences, the essay ends up offering a less than fully satisfactory treatment of familiar and, in principle, remediable issues, including vague or insufficiently examined constructs, overly rigid explanatory standards, unwarranted expectations placed on statistical models, and the tendency to treat population-level regularities as individual-level explanations. Moreover, this treatment is accompanied by broader considerations about how close we are to The Truth or how deep our understanding runs—metaphysical concerns that distract from the very material issues at hand. In our view, a more fruitful perspective on these issues is obtained through the lens of a hierarchy of models in which contact between theory and the world is mediated by a sequence of representational steps, each with its own standards of adequacy and opportunities for error (Kellen, 2019; Mayo, 1996; Suppes, 1966). When difficulties arise, the question is not where The Truth lies but which step in the chain of representation has failed.

## Supplementary Information

Below is the link to the electronic supplementary material.

Supplementary file 1 (pdf 607 KB)

## Data Availability

Not applicable.

## Footnotes

1 An earlier, extended version is available at https://doi.org/10.31234/osf.io/s5ept_v1.

2 The conflation is evident in the unfortunate phrasing, “Mathematical or computer simulation models are often proposed as causes of a pattern of data.” Models, as formal representations of explanations, may of course portray causal claims, but the model itself cannot be a cause of anything, let alone data. To suggest otherwise would be like claiming that the addition operator ‘+’ *causes* 2 + 2 to be 4.
