# Supplementary material for: Scientific Misunderstanding: Notes for the Bedeviled
Source: Comput Brain Behav. 2026 Sep 15;9(3):401–4. doi: 10.1007/s42113-026-00337-0 (PMC13594071; doi:10.1007/s42113-026-00337-0)
Supplement: Supplementary file 1 [file 42113_2026_337_MOESM1_ESM.pdf]

Supplemental Materials

This Supplemental Material has two goals. The first is to offer a proof of concept of how three seemingly conflicting claims in fact coexist without tension and without appeal to regression to the mean (RTM): (i) Statistician 1's conclusion of no mean difference in weight change between the sexes; (ii) Statistician 2's finding that, for a given initial weight, boys gain more than girls; and (iii) the essay's observation that, conditional on a given *final* weight, boys must have been initially lighter than girls, yielding what appears to be a reversed comparison.

The second goal is to spell out the implausible claims about measurement error and transient variation that the regression to the mean account, annointed by the essay but never scrutinized, is forced to make in order to account for simple scenarios where modest effects are found.

Starting wit the first goal, let $W_{0,i}$ denote initial weight and $W_{1,i}$ final weight of child i. Let $S_i \in \{\mathrm{G}, \mathrm{B}\}$ denote the sex of the child with $S_i = \mathrm{G}$ for girls and $S_i = \mathrm{B}$ for boys. Define the time-forward change in weight as $\Delta_i = W_{1,i} - W_{0,i}$. Moreover, let $\mu_{\mathrm{G}} = \mathbb{E}[W_0 \mid S = \mathrm{G}]$ and $\mu_{\mathrm{B}} = \mathbb{E}[W_0 \mid S = \mathrm{B}]$ denote the mean initial weights for girls and boys, respectively, and assume $\mu_{\mathrm{B}} > \mu_{\mathrm{G}}$.

Now, let us suppose that forward weight change is governed by the linear model

$$\Delta_i = \alpha + \beta s_i - \gamma W_{0,i},$$

where α represents an overall level of weight change, β corresponds to a sex difference in weight change, s represents an indicator variable for sex that takes on $s_i = 1$ if $S_i = \mathrm{B}$, and γ captures the dependence of change on initial weight. Assume $0 < \gamma < 1$, so that weight change is self-limiting (i.e., the ability to gain weight diminishes for greater initial weights). To keep the algebra as clean as possible, we're purposely omitting individual-level residuals without loss of generality. We are also making no appeal to individual-level systematic effects (another potential avenue for describing Lord's Paradox) as these are not identifiable in the experimental design considered by

Lord (1967).

We first recover Statistician 2's claim. Holding initial weight fixed at a common value for everyone, $W_0 = x$, we have

$$\mathbb{E}[\Delta \mid W_0 = x, s] = \alpha + \beta s - \gamma x,$$

which means that

$$\mathbb{E}[\Delta \mid W_0 = x, s = 1] - \mathbb{E}[\Delta \mid W_0 = x, s = 0] = \beta.$$

In words, boys are expected to gain more than girls with the same initial weight whenever $\beta > 0$.

Next, let us show how Statistician 1's conclusion arises in this model. Taking expectations within each group yields

$$\mathbb{E}[\Delta \mid s = 0] = \alpha - \gamma \mu_{\mathrm{G}},$$
$$\mathbb{E}[\Delta \mid s = 1] = \alpha + \beta - \gamma \mu_{\mathrm{B}}.$$

If we set

$$\alpha = \gamma \mu_{\mathrm{G}}, \qquad \beta = \gamma(\mu_{\mathrm{B}} - \mu_{\mathrm{G}}),$$

then

$$\mathbb{E}[\Delta \mid s = 0] = 0,$$
$$\mathbb{E}[\Delta \mid s = 1] = 0.$$

In words, both groups exhibit no net change in weight, reproducing Statistician 1's conclusion.

Crucially, this result coexists alongside the positive conditional difference identified earlier.

Finally, we derive the reversed comparison emphasized in the essay. From the equation for final weight,

$$W_{1,i} = \alpha + (1 - \gamma)W_{0,i} + \beta s_i,$$

solve for initial weight:

$$W_{0,i} = \frac{W_{1,i} - \alpha - \beta s_i}{1 - \gamma}.$$

Holding final weight fixed at value $W_1 = y$, we obtain

$$\mathbb{E}[W_0 \mid W_1 = y, s = 1] = \frac{y - \alpha - \beta}{1 - \gamma},$$
$$\mathbb{E}[W_0 \mid W_1 = y, s = 0] = \frac{y - \alpha}{1 - \gamma}.$$

It follows then that

$$\mathbb{E}[W_0 \mid W_1 = y, s = 1] - \mathbb{E}[W_0 \mid W_1 = y, s = 0] = -\frac{\beta}{1 - \gamma} < 0,$$

meaning that, assuming the same final weight, boys must have started at a lower initial weight than girls. This is the reversal noted by the essay when conditioning on final rather than initial weight.

To make all of this more concrete, let us consider an illustrated example: Let $\mu_{\text{G}} = 120$, $\mu_{\text{B}} = 150$, and $\gamma = 0.2$. As before, we're fixing $\alpha = \gamma\mu_{\text{G}}$ and $\beta = \gamma(\mu_{\text{B}} - \mu_{\text{G}})$. To make things more realistic-looking, we added individual-level noise to both initial and final weights. The noise terms were independent random samples from a normal distribution with a mean of zero and standard deviation of 20. We generated 2000 samples per sex. As shown in Figure 1, the resulting data closely match the Lord (1967) example (which is also illustrated in the essay).

Taken together, the results arise from the same linear system under three distinct com-

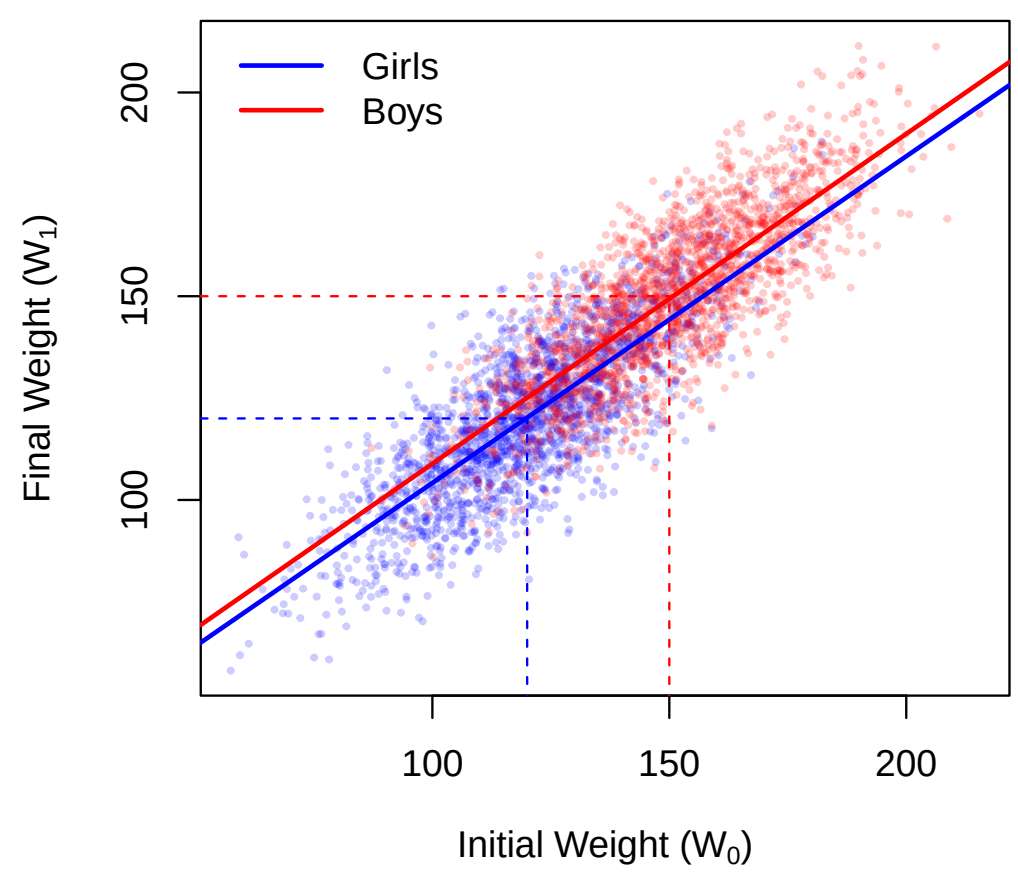

Figure 1
Illustration of Lord's Paradox Using the Proposed Model

parisons: (i) averaging over group-specific baseline distributions, (ii) holding initial weight fixed, and (iii) holding final weight fixed. Therefore, there is no real tension between the three claims. The proposed model achieves this by positing a simple tradeoff: On one hand, boys see a positive increment in weight gain relative to comparable girls, but they also begin heavier. On the other, the weight-gain process is self-limiting, such that higher starting values diminish potential subsequent increases. Note that RTM is neither involved nor necessary. The two forces posited by the model pull in opposite directions and can be finetuned to cancel in the aggregate, as done here. The same system can yield no mean difference across groups while preserving positive differences at fixed baselines. The rest boils down to bookkeeping about what is held fixed: Condition on the initial weight, and a sex-difference in weight increment is observed. Condition on final weight, and the baseline disparity reasserts itself through the statement that, to reach the same endpoint, boys must have started lower. These are simply alternative perspectives on the same joint distribution.

We now turn to our second goal, which is to make explicit what is required of a RTM account in order to handle even modest conditional effects.

Under an RTM account with individual heterogeneity, initial and final weights are taken to be noisy measurements of a stable true weight. Keeping with the notation above, let

$$W_{0,i} = \mu_{S_i} + U_i + \varepsilon_{0,i}, \qquad W_{1,i} = \mu_{S_i} + U_i + \varepsilon_{1,i},$$

where μ_{S_i} denotes the sex-specific mean, U_i is a stable individual deviation with $\mathbb{E}[U_i \mid S_i] = 0$, and $\varepsilon_{0,i}$ and $\varepsilon_{1,i}$ are measurement errors or transient fluctuations following independent zero-centered Normal distributions.

Skipping a few dull steps here, the RTM account can be shown to imply that the conditional difference identified by Statistician 2 is

$$\mathbb{E}[\Delta \mid W_0 = x, s = 1] - \mathbb{E}[\Delta \mid W_0 = x, s = 0] = \theta(\mu_{\text{B}} - \mu_{\text{G}}),$$

where

$$\theta = \frac{\text{var}_{\text{error}}}{\text{var}_{\text{error}} + \text{var}_{\text{ind}}}.$$

This result shows that the magnitude of the conditional effect is entirely determined by the group difference $\mu_{\text{B}} - \mu_{\text{G}}$ and the relative contribution of measurement error quantified by θ.

To illustrate, consider the same numerical setting used in Figure 1, where $\mu_{\text{G}} = 120$, $\mu_{\text{B}} = 150$, and the within-sex standard deviation in true weights is 20 (i.e., $\text{var}_{\text{ind}} = 400$). Now, suppose that the conditional difference $\mathbb{E}[\Delta \mid W_0 = x, s = 1] - \mathbb{E}[\Delta \mid W_0 = x, s = 0]$ is 6. The RTM expression then implies $\theta = 0.2$, which requires that $\text{var}_{\text{error}} = 100$, which in turn entails a standard deviation of measurement error of 10 lbs! This means that, in order to account for the same results, RTM must assume an implausible amount of measurement error and transient fluctuations in body weight.
